# Only vulnerable adults show change in chronic low-grade inflammation after contemplative mental training: evidence from a randomized clinical trial

**DOI:** 10.1038/s41598-019-55250-3

**Published:** 2019-12-18

**Authors:** Lara M. C. Puhlmann, Veronika Engert, Filia Apostolakou, Ioannis Papassotiriou, George P. Chrousos, Pascal Vrtička, Tania Singer

**Affiliations:** 1 Research Group “Social Stress and Family Health”, Max Planck Institute for Human Cognitive and Brain Sciences, Leipzig, Germany; 2 Department of Clinical Biochemistry, “Aghia Sophia” Children’s Hospital, Athens, Greece; 3 First Department of Pediatrics, National and Kapodistrian University of Athens Medical School, “Aghia Sophia” Children’s Hospital, Athens, Greece; 4 Social Neuroscience Lab, Max Planck Society, Berlin, Germany; 5 Department of Social Neuroscience, Institute of Psychosocial Medicine and Psychotherapy, University Hospital, Jena, Germany

**Keywords:** Cytokines, Human behaviour, Predictive markers

## Abstract

Growing evidence suggests that chronic low-grade inflammation can be reduced through mindfulness-based mental training interventions. However, these results are inconsistent and based on patient populations with heterogeneous conditions. Similar research in healthy adults is lacking. Moreover, common intervention protocols involve varying combinations of different contemplative practices, such that it remains unclear which types of training most effectively influence biomarkers of inflammation. The present study investigated the effect of three distinct 3-month training modules cultivating a) interoception and present-moment focus (Presence), b) socio-affective skills (Affect), or c) socio-cognitive skills (Perspective) on the inflammatory biomarkers interleukin-6 (IL-6) and high sensitive C-reactive protein (hs-CRP) in 298 healthy adults. We observed no group-level effect of training on either biomarker, but trend-level interactions of training type and participant sex. In additionally exploring the influence of participants’ baseline inflammation, a selective training effect emerged: Following the Presence module, participants with relatively higher inflammatory load showed stronger reduction in IL-6 on average, and in hs-CRP if they were male. Mindfulness- and attention-based mental practice thus appears most effective when targeting chronic low-grade inflammation in healthy adults, particularly in men. Overall, our data point to a floor effect in the reduction of inflammatory markers through contemplative mental training, suggesting that mental training may be less effective in improving basal biological health outcomes in healthy, low-stressed adults than in vulnerable populations.

## Introduction

Contemplative mental training interventions have become popular non-pharmacological treatment options with the potential to promote mental and physiological well-being in healthy and clinical populations^1–3^. The majority of intervention protocols are based on mindfulness^4^, which has its roots in the contemplative traditions of Buddhism and has been described as “paying attention in a particular way; on purpose, in the present moment and non-judgmentally”^5^. To better understand the physiological processes that presumably underlie the beneficial effects of such interventions, a growing body of research investigates how subjectively reported improvements in well-being relate to changes in biological markers of health, such as inflammatory proteins^6,7^. However, observed effects on biomarkers of inflammation are inconsistent to date^8,9^. Here, we aimed to achieve more reliable results through a large-scale intervention with a healthy, homogeneous subject population tested in a differential intervention protocol.

The effect of contemplative mental training on inflammatory biomarkers has gained particular interest because the immune system is implicated in a wide range of prevalent mental and physiological conditions, including cardiometabolic disease, depression, and cancer^10–12^. The most commonly assayed biomarkers of inflammation include the pro-inflammatory cytokine interleukin-6 (IL-6), and the surrogate marker of low-grade inflammation high sensitive C-reactive protein (hs-CRP). Both have been linked reliably to clinical conditions involving the immune system^10,13^. Elevated IL-6 or hs-CRP levels in the absence of an acute infection or other inflammatory stimuli signal the presence of maladaptive chronic low-grade inflammation^14^, while lower levels are associated with better physical health and well-being^15^.

Almost all studies investigating the effects of mental training on inflammatory parameters focus either on patients or at-risk participants. Several mindfulness-based interventions with cancer patients and groups at risk for developing clinical conditions, such as older adults, found evidence for reduced basal concentrations of IL-6^16–18^ and hs-CRP^19,20^. However, reviews have highlighted that these effects are not found consistently^8,9,21^. The various ailments of participants are a major source of heterogeneity between investigations that likely contributes to these inconsistencies. Research with healthy populations avoids this issue and may eventually yield more generalizable results. Notably, subclinical elevations in IL-6 and hs-CRP are predictive markers for conditions such as coronary heart disease^22^ and type 2 diabetes^23^. Lowering them is therefore of interest even for healthy individuals to prevent the onset of disease. Research in healthy participants has been neglected, however. In part, the predominant focus on at-risk groups may be explained by considerations of study power: in the still emerging field of contemplative science, relatively short intervention durations and limited sample sizes create a framework were only medium to strong effects can be detected. To achieve sufficient power, researchers may have focused on at-risk groups, for whom larger intervention effects can be expected.

Current health outcomes in at-risk groups are most prominently explained through a stress buffering account. Creswell and Lindsay (2014) for example propose that training effects are mediated through a reduction in experienced stress and lower activity in associated physiological pathways^6^. If stress is the key mediator, mental training may predominantly benefit patients whose conditions are exacerbated by stress, or healthy but highly stressed individuals. The health outcomes of low-risk participants, i.e. healthy and not selected for vulnerabilities such as chronic stress, may not be improved. Evidence from acute stress situations, however, paints a different picture: Following mental training, healthy participants showed reduced immune reactivity in response to an acute psychosocial stress induction^24,25^. In an acute context, one can expect larger effect sizes, similar to research with at-risk populations. The consequently higher statistical power may explain why, to date, training effects on IL-6 and hs-CRP in healthy participants have been found specifically in acute settings. Therefore, in order to reliably distinguish whether mental training interventions have either no effect or small effects on basal IL-6 and hs-CRP levels in low-risk adults, a sufficiently powered large-scale intervention study is necessary. To our knowledge, there is no published investigation that fulfills this requirement. Pace and colleagues (2009) reported one null finding regarding basal IL-6 levels, but in a relatively small sample of 61 healthy college students and after a specifically compassion-focused training protocol, limiting generalizability^25^. Studies using mind-body therapies (MBTs) that involve physical exercises such as Qi Gong have also produced mixed results^26,27^. To avoid conflation with exercise-related effects on inflammatory markers^28,29^ these should not be equated with strictly mental training protocols.

Next to differences in the studied population, inconsistency in the literature also stems from the use of different intervention protocols. Mindfulness-based training protocols usually include several different types of contemplative practices, making it difficult to infer which practice specifically contributes to an observed effect. Recent categorizations have proposed three different classes of mental practices that focus either on the cultivation of attention, the active generation of positive social affect such as compassion, or on analytical cognitive skills and perspective taking^30–32^. Established protocols primarily train mindfulness practices focused on present-moment awareness and attention, such as in the 8-week mindfulness-based stress reduction program (MBSR)^33^ and the mindfulness-based cognitive therapy program (MBCT)^34^. Newer intervention designs prioritize the explicit cultivation of affective qualities, including loving kindness and (self-)compassion, for example through compassion-focused therapy (CFT)^35^ or the 8-week mindful self-compassion program (MSC)^36^. Compared to mindfulness-based interventions, very few studies have investigated how compassion-based interventions affect serum IL-6 and hs-CRP levels, with mixed results but some success^25,37^. A comprehensive investigation of the effect of mental training on IL-6 and hs-CRP should include both of these intervention types, in order to allow conclusions not only about the effectiveness of specific practices, but about contemplative mental training more generally.

**Figure 1:**
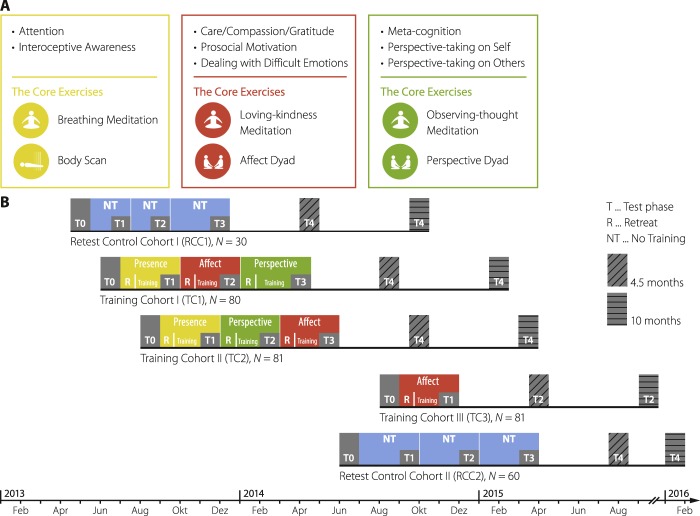
Study design of the ReSource Project. (**A**) central processes and core exercises trained during the three modules Presence (yellow), Affect (red), and Perspective (green). (**B**) timeline of the ReSource Project and training sequences per cohort. Colored areas represent training periods and grey areas data collection. The study timeline was adapted to most accurately reflect blood sampling; test phases for other variables may differ slightly. For logistic reasons retest control participants were recruited in two cohorts, but are analyzed jointly. The full ReSource design as shown in the Figure contained follow up assessments (T4); however, these are not included in the present investigation. Figures were adapted with permission from Singer *et al*. (2016).

The current study aimed at disentangling the differential effects of three distinct types of contemplative mental training on chronic low-grade inflammation in the context of the ReSource Project, a large-scale longitudinal intervention study with 332 healthy adults. Participants practiced a sequence of three different three-month training modules, each cultivating distinct mental capacities (see Fig. 1): The Presence module trained present-moment-focused awareness, attention, and interoception. These capacities are also central to MBSR and MBCT protocols, such that Presence is best comparable to classic mindfulness-based intervention programs. The Affect module focused on cultivating socio-affective qualities such as loving kindness, gratitude, and compassion, and is therefore comparable to compassion-based therapies such as the CFT or MSC. Finally, the Perspective module aimed at increasing socio-cognitive and meta-cognitive analytical abilities. To our knowledge, there is no literature on training protocols that are comparable to the Perspective module and their effect on inflammatory markers. Since our primary aim was to extend on previous inconsistent results from patient-population based studies, potential effects of the Perspective training were not part of our main hypotheses. More specific hypotheses for the Perspective training exist for other investigations in the context of the ReSource Project.

We additionally explored the influence of individual differences, which are often neglected in contemplative training studies^38^. Sex-differences are known to influence immune system activity^39,40^ and were therefore a factor of interest in the present investigation. Initial inflammatory load, measured by baseline levels of IL-6 and hs-CRP, was considered an indicator for whether, and to what extent, participants were at risk of developing clinical conditions. Finally, measures of chronic stress were included as potential mediators of training effects.

Based on the available literature, we primarily expected that circulating levels of IL-6 and hs-CRP would decrease significantly after the Presence and the Affect training compared to a no-training retest condition. Perspective was also anticipated to reduce both biomarkers, possibly through its regulatory influence on participants stress responses^41^. We further investigated how participants’ baseline inflammation levels would influence their response to the training in exploratory follow-up analyses, to examine how mental training interacted with participants’ vulnerability or risk-status.

## Methods

### Participants

An initial sample of N = 332 ostensibly healthy participants aged 20–55 were recruited in the context of the ReSource Project^42^ and assigned to one of three training cohorts (TC1, N = 80; TC2, N = 81; TC3, N = 81) or to a retest control cohort (RCC, N = 90). Participants of the RCC did not receive any training but underwent the same testing procedures as participants of the TCs. Cohort assignment was done through bootstrapping without replacement to ensure the formation of demographically homogeneous groups. The resulting allocation ratio was approximately 1:1:1:1. From all eligible participants, the final sample selection and size was determined by two constrains: the formation of demographically homogenous cohort groups, and feasibility of group-based training. For further detail on the selection of participants and the CONSORT flow diagram, see Singer *et al*., 2016, chapter 7, pp. 48–49. All participants were meditation-naïve and extensively screened on mental health questionnaires. Additionally, they underwent clinical diagnostic interviews with a trained psychologist (Structured Clinical Interview for DSM-IV Axis-I (SCID-I)^43^; SCID-II for Axis-II disorders^44^) and were excluded if they fulfilled the criteria for an Axis-I disorder within the past two years or if they fulfilled the criteria for an Axis-II disorder at any point in their life. For an extensive description of baseline demographic characteristics, see Singer *et al*., 2016, Appendix C2.

All participants gave written informed consent prior to participation, could withdraw from the study at any time and were financially compensated. The study was approved by the Research Ethics Committee of the University of Leipzig (ethic number: 376/12-ff) and the Research Ethics Committee of the Humboldt University in Berlin (ethic numbers: 2013-02, 2013-29, 2014-10). It was registered under the title “Plasticity of the Compassionate Brain” with the Protocol Registration System of ClinicalTrials.gov (Identifier: NCT01833104; date of registration: 16/04/2013). All methods were performed in accordance with the above protocols and with the Declaration of Helsinki.

### Training intervention

Participants of the TCs were taught in three distinct three-month long modules termed Presence, Affect, and Perspective. Each module began with a 3-day retreat, during which professional teachers introduced participants to the conceptual core and the relevant practices of a given module. Afterwards, participants attended weekly 2-hour group sessions and exercised the two core practices of each module at home for approximately 30 minutes daily, supported by a tailor-made app and online platform. Figure 1A shows the concepts and core practices of the three modules. The Presence module trained present-moment-focused awareness, attention, and interoception using the classic meditation techniques Body Scan and Breathing Meditation as daily core practices. These mindfulness-based practices are also central to the well-established training protocols MBSR and MBCT. The Affect module focused on cultivating qualities of the heart such as loving kindness, gratitude, compassion, and accepting difficult emotions. Daily core practices of this socio-affective module were a Loving-Kindness Meditation and a specific Affect Dyad. The Affect Dyad is a partner exercise that was designed to develop empathy, gratitude, compassion, and non-judgmental acceptance of difficult emotions. The Perspective module aimed at increasing socio-cognitive and meta-cognitive analytical abilities. Its daily core practices included the classical Observing-Thoughts Meditation, which is also part of many mindfulness-based interventions such as MBSR and MBCT, and the Perspective Dyad. This partner exercise trained cognitive perspective taking on the beliefs and thoughts of oneself and others. See also Singer *et al*. (2016), chapter 3.

### Study design

All training and data collection took place between April 2013 and February 2016. The study followed a mixed design, in which most but not all participants received all types of training. The sequence of training modules was varied across the three TCs for the purpose of counterbalancing sequence effects. Two TCs completed all modules in the order Presence, Affect, Perspective (TC1) or Presence, Perspective, Affect (TC2), for a total of nine months of training. A third cohort underwent three months of Affect only training (TC3), with the aim of isolating the effects of the Presence module. The TCs thus served as active control groups for one another and allowed to identify if two modules that were performed at the same time but differed in content had distinguishable effects (see also Fig. 1B and Singer *et al*., 2016, chapter 4). The trial was ended after completion.

### Biological assays

For the assessment of IL-6 and hs-CRP, 5.5 ml blood was collected from each participant into serum vacutainers (Sarstedt, Nümbrecht, Germany) at time points T0-T3, i.e., at baseline and after the completion of each 3-month training module or time interval in the case of the RCC. To control for diurnal fluctuations in biomarkers, a single participant provided their samples always at the same time of day (mean deviation in sampling time (SD): −0.087 (2.30) hrs). Sampled blood was allowed to clot for 30–45 minutes and subsequently centrifuged at 3500 RPM for 15 minutes. The resulting serum was frozen at −80 °C until assay, which was performed at the Department of Clinical Biochemistry at “Aghia Sophia” Children’s Hospital, Athens, Greece. IL-6 levels were measured in picogram per milliliter (pg/ml) using a solid-phase, two-site chemiluminescence immunometric assay on the Siemens IMMULITE® 2000 immunoassay analyzer (Siemens Healthineers Tarrytown, NY, USA) according to the manufacturer’s instructions. The detection limit was 2 pg/ml and the inter-assay coefficients of variation lay between 3.9 and 14.3%, depending on the sample concentration. hs-CRP concentrations, measured in milligram per liter (mg/L), were assessed with the high-sensitivity-CRP (hs-CRP) method of latex-enhanced immunoturbidimetric assay, using the Siemens Advia 1800 Clinical Chemistry System (Siemens Healthineers Tarrytown, NY, USA). The low-end precision performance was 0.16 mg/L, and the inter-assay coefficients of variation were 5.3% and 6.8%, respectively. Samples of both IL-6 and hs-CRP were thus continuously loaded and analyzed as opposed to being run on plates, resulting in very low inter and intra assay coefficients of variation. No sample contained hs-CRP concentrations under the detection limit.

### Questionnaires and lifestyle variables

Long-term stress was measured using two self-report questionnaires, the Trier Inventory for Chronic Stress (TICS)^45^ and the Perceived Stress Scale (PSS)^46^. Participants completed both questionnaires at time points T0–T3. The TICS comprises 39-items which measure six facets of chronic stress (work overload, worries, social stress, lack of social recognition, work discontent and intrusive memories) and one global stress score, each capturing a time span of 1–3 months. The 10-item PSS measures to which extent situations in the past month of one’s life were experienced as unpredictable, uncontrollable, and overloaded. It is the most widely used psychological instrument for measuring the perception of stress and produces one summary stress score. Both questionnaires have satisfactory reliability and validity^45,46^. Moreover, three self-report items describing change in health-related behavior were analyzed to control for the influence of physical exercise, sleep, and healthy eating on chronic low-grade inflammation^28,29^. Behavioral change was recorded from participants of the TCs but not the RCC following the completion of each training module (T1–T3). Participants reported how much they performed physical exercise, slept, and ate healthily in the most recent compared to the previous training phase on a 5-point Likert scale ranging from -2 (“much less”) to +2 (“much more”); see also Singer *et al*. (2016), Appendix H. Finally, practice frequency was recorded via the tailor-made training platform.

### Statistical analyses

#### Preprocessing of dependent variables

Raw IL-6 and hs-CRP values were treated with a natural log transformation to remedy high kurtosis that would lead to non-normal distributions of model residuals if untreated. Subsequently, IL-6 and hs-CRP values for the main analyses were calculated as difference scores between each participant’s ln-transformed measurements from a set of consecutive time points (T1-T0, T2-T1, and T3-T2). Difference scores allow modeling change directly as a function of the training module that each participant practiced in the respective time interval. This approach was also chosen to avoid biasing the analysis by including different subjects before and after a given time point. The calculated difference scores were checked for outliers defined as values diverging more than three standard deviations from the sample mean. Outliers were winsorized to the respective lower and upper boundaries of three standard deviations. TICS and PSS change scores were calculated and outlier-corrected following the same steps. While some previous studies have suggested that CRP values >10 mg/L correspond to an acute infection and should be excluded in the analysis of chronic low-grade inflammation, this cut-off approach has been criticized for being arbitrary if researchers do not have additional information about a participants acute health status^47^. Such additional health-related information was not available in the ReSource Project. To avoid censoring the data we therefore opted to use all available data points.

#### Statistical models for planned analyses

Statistical analyses were conducted and visualized in IBM SPSS Statistics version 24 (IBM Corp., Armonk, N.Y., USA) and R version 3.5.1^48^, respectively. Table 1 shows all variables selected for statistical analyses and their conceptual relevance to the planned analyses. Body-mass index (BMI) and age were included as covariates and sex as a factor in all analyses to control for their potential influence on serum levels of IL-6 and/or hs-CRP^8,29^. Change analyses were conducted using linear mixed models (LMMs), which are robust to unbalanced and incomplete data in longitudinal designs. For each LMM, the factors time, module, and sex were entered as fixed effects, and time additionally as repeated factor with autoregressive covariate structure AR(1). The term ‘module’ indexed each training type and was used to analyze the effect of all three training modules and no training on IL-6 and hs-CRP. ‘Module’ was therefore the main variable of interest in each LMM. A significant effect of the term module would suggest that IL-6/hs-CRP changed differentially depending on the practiced training. Time and module were also interacted to investigate whether the effect of each module was stable over all time points (see ‘Analysis of sequence effects’ below). To allow module effects to differ non-linearly between time points, time was structured as a categorical rather than continuous variable. To account for the potential moderating influence of sex-differences on training effects, sex was interacted with the factors time and module. Baseline levels of either IL-6 or hs-CRP were included as a continuous between-subject covariate in analyses targeting change scores of the same marker. A random intercept for subject ID was included in all LMMs. The full planned LMMs therefore included the following terms:

$$\begin{array}{ccc}\Delta {\rm{I}}{\rm{L}} \mbox{-} {6}_{{\rm{i}}{\rm{j}}}/\Delta {\rm{h}}{\rm{s}} \mbox{-} {{\rm{C}}{\rm{R}}{\rm{P}}}_{{\rm{i}}{\rm{j}}} & = & {\beta }_{0}+{\beta }_{1}{{\rm{a}}{\rm{g}}{\rm{e}}}_{{\rm{i}}}+{\beta }_{2}{{\rm{B}}{\rm{M}}{\rm{I}}}_{{\rm{i}}}+{\beta }_{3}{{\rm{s}}{\rm{e}}{\rm{x}}}_{{\rm{i}}}+{\beta }_{4}{{\rm{T}}{\rm{I}}{\rm{C}}{\rm{S}}}_{{\rm{i}}{\rm{j}}}+{\beta }_{5}{{\rm{P}}{\rm{S}}{\rm{S}}}_{{\rm{i}}{\rm{j}}}\\  &  & +{\beta }_{6}\,{\rm{I}}{\rm{L}} \mbox{-} 6\,/\,{\rm{h}}{\rm{s}} \mbox{-} {\rm{C}}{\rm{R}}{\rm{P}}{{\rm{b}}{\rm{a}}{\rm{s}}{\rm{e}}{\rm{l}}{\rm{i}}{\rm{n}}{\rm{e}}}_{{\rm{i}}}\,+{\beta }_{7\text{-}9}{{\rm{m}}{\rm{o}}{\rm{d}}{\rm{u}}{\rm{l}}{\rm{e}}}_{{\rm{i}}{\rm{j}}}+{\beta }_{10\text{-}11}{{\rm{t}}{\rm{i}}{\rm{m}}{\rm{e}}}_{{\rm{j}}}\\  &  & +{\beta }_{12\text{-}16}\,{{\rm{m}}{\rm{o}}{\rm{d}}{\rm{u}}{\rm{l}}{\rm{e}}}_{{\rm{i}}{\rm{j}}}\times {{\rm{t}}{\rm{i}}{\rm{m}}{\rm{e}}}_{{\rm{j}}}+{\beta }_{17\text{-}19}{{\rm{m}}{\rm{o}}{\rm{d}}{\rm{u}}{\rm{l}}{\rm{e}}}_{{\rm{i}}{\rm{j}}}\times {{\rm{s}}{\rm{e}}{\rm{x}}}_{{\rm{i}}}\\  &  & +{\beta }_{20\text{-}21}{{\rm{t}}{\rm{i}}{\rm{m}}{\rm{e}}}_{{\rm{j}}}\times {{\rm{s}}{\rm{e}}{\rm{x}}}_{{\rm{i}}}+{\beta }_{22\text{-}26}{{\rm{m}}{\rm{o}}{\rm{d}}{\rm{u}}{\rm{l}}{\rm{e}}}_{{\rm{i}}{\rm{j}}}\times {{\rm{t}}{\rm{i}}{\rm{m}}{\rm{e}}}_{{\rm{j}}}\times {{\rm{s}}{\rm{e}}{\rm{x}}}_{{\rm{i}}}+{\rm{r}}{\rm{a}}{\rm{n}}{\rm{d}}{({\rm{I}}{\rm{D}})}_{{\rm{i}}},\end{array}$$

where i = subject, j = time point of measurement, ΔIL-6 = change in IL-6, and Δhs-CRP = change in hs-CRP.

**Table 1:** Source and relevance of variables selected for statistical analysis.

Measure	Indicator of	Assessment method	Relevance
IL-6, hs-CRP	Chronic inflammation	Blood	Primary outcome measure
IL-6/hs-CRP levels at T0	Subject-specific inflammatory load/risk status	Blood	Potential predictor of individual responsiveness to training
Sex	Sex-differences	Self-report	Indicator of sex-specific training effects and steroid effects on IL-6/hs-CRP
Age	Physical constitution	Self-report	Potential confounding variable
BMI	Physical constitution	Physical assessment	Potential confounding variable
TICS, PSS	Chronic stress	Self-report	Potential mediator of training effect
Change in physical exercise, sleep, healthy diet	Health-related behavior	Self-report	Potential confound for training effect
N core exercises practiced	Practice intensity	Tracking through online platform	Potential modulator of training effect

*Note:* The four columns outline which larger constructs we aimed to assess (“Indicator of”), which measures were used for this purpose (“Measures”), through which method these measures were assessed (“Assessment method”), and their conceptual relevance to the planned analyses (“Relevance”). BMI denotes body mass index; IL-6, interleukin-6; hs-CRP, high sensitive C-reactive protein; TICS, Trier Inventory for Chronic Stress; PSS, Perceived Stress Scale.

TICS and PSS change scores were evaluated in two steps. First, both questionnaires were included as continuous within-subject covariates in the main models to assess the influence of individual change in experienced stress on IL-6 and hs-CRP fluctuations in general. Second, we evaluated the role of self-reported stress for any significant effect of one or several training modules on IL-6 or hs-CRP by selectively regressing biomarker change onto questionnaire change scores for each relevant module. The variables practice frequency and change in health-related behavior could not be included in the main LMMs since they were only available from the training participants. Instead, their role in any potential training effects was analyzed in targeted regression analyses following the same procedure as for TICS and PSS scores.

#### Analysis of sequence effects

Our main term of interest was module, which indicated the practiced training type (i.e., no training, Presence, Affect and Perspective). In each analysis, we first examined interactions of the terms time and module to identify whether module effects depended on the time at which they were practiced (i.e. as first module or after three or six months of other contemplative training). A significant interaction would suggest that the training sequence influenced the effect of one or several modules. Conversely, the absence of an interaction with time would indicate that module effects did not significantly differ as a function of the training sequence and may therefore be combined. In the latter cases, we maximized analysis power by comparing the estimated average change following all training instances of the same module.

Even in the absence of a significant module x time interaction, the comparability of average effects can be compromised by the fact that the three training modules were practiced at different time points and with differing durations of prior training. Given a significant effect of one or several modules on average, we investigated this potential confound by plotting estimates from the module by time interaction, which differentiates each training interval. Follow-up *t*-tests were conducted to assess if module differences that were significant on average remained similarly sized and robust when compared only to modules practiced at the same time interval.

#### Effect sizes and follow-up analyses

Effect sizes of the variables of interest that were significant in the LMMs were calculated as omega squared (ω^2^), by dividing the variance of the residuals of the full model by the variance of residuals of a model without the variable of interest, and subtracting the outcome from 1^49^. The resulting effect sizes were classified as small (ω^2^ ≥ 0.010), medium (ω^2^ ≥ 0.059), or large (ω^2^ ≥ 0.138)^50^. Significant main effects of, or interactions with categorical variables of interest were decomposed within the multilevel model framework through follow-up Student *t*-tests, which directly contrasted model estimates. Since these contrasts were conducted as part of the main LMMs, they were not corrected for multiple comparisons.

## Results

### Raw and missing data

Table 2 shows the raw scores of all variables of interest per training module. The final sample consisted of N = 688 sets of measurements with all covariates, obtained from N = 296 participants. Supplementary Tables describe the sample sizes and reasons for missing cases (Tables S1, S2) as well as raw change in IL-6, hs-CRP, TICS, and PSS (Table S3).

**Table 2:** Raw mean (SD) of all variables of interest per training module.

	No training^a^	Presence^b^	Affect^b^	Perspective^b^
N (females)	197 (117 f)	143 (85 f)	211 (122 f)	137 (77 f)
Age /years (SD)	39.6 (9.3)	41.0 (9.5)	40.9 (9.4)	41.0 (9.6)
BMI (SD)	23.8 (3.1)	23.6 (3.3)	23.4 (3.1)	23.6 (3.2)
IL-6 pg/ml (SD)	1.53 (0.23)	1.51 (0.25)	1.61 (0.62)	1.60 (0.69)
hs-CRP mg/L (SD)	1.29 (1.68)	1.42 (2.08)	1.38 (1.89)	1.52 (2.15)
TICS global stress (SD)	14.8 (7.96)	13.5 (7.26)	13.7 (7.78)	12.1 (7.32)
PSS total (SD)	13.9 (5.72)	13.6 (5.76)	12.9 (6.41)	11.4 (6.00)
**Sampling time**				
hrs after 00:00 (SD)	14.12 (3.56)	14.17 (3.28)	14.17 (3.48)	13.26 (3.31)
Exercise change (SD)	—	−0.09 (0.80)	0.06 (0.71)	0.09 (0.76)
Sleep change (SD)	—	0.05 (0.67)	0.03 (0.66)	0.03 (0.63)
Diet change(SD)	—	0.25 (0.56)	0.21 (0.56)	0.31 (0.65)
Practice N/week:				
Meditation (SD)	—	4.79 (1.16)	3.85 (1.34)	3.65 (1.19)
Dyad (SD)	—	—	3.83 (0.67)	3.53 (0.68)

^a^No training includes repeated observations at T1, T2 and T3 from the n = 76 (46 f) retest control cohort participants.

^b^Presence, Affect, and Perspective each include unique observations from the training cohort participants, n = 149 TC1 & TC2 (88 f); n = 71 TC3 (41 f).

*Note:* Summary statistics in this table were calculated based on the final sample of participants with all covariates of interest. Mean raw values following the respective modules (no training, Presence, Affect, Perspective) are shown. For an extensive description of baseline demographic characteristics, see Singer *et al*., 2016, Appendix C2. BMI denotes body mass index; IL-6, interleukin-6; hs-CRP, high sensitive C-reactive protein; TICS, Trier Inventory for Chronic Stress; PSS, Perceived Stress Scale; SD, standard deviation. See Supplementary Table S3 for IL-6, hs-CRP, TICS and PSS difference scores per time point and module.

### Baseline analyses

Table S4 shows the results of initial correlational analyses performed with baseline IL-6 and hs-CRP data as well as all covariates available at T0. Univariate analyses revealed significant sex-differences in IL-6 (*F*(1,303) = 4.85, *p* = 0.028) and hs-CRP (*F*(1,303) = 11.3, *p* = 0.001). Females showed higher concentrations of, and higher variance in both biomarkers (raw mean (SD), IL-6 females: 1.77 (2.02) pg/ml, IL-6 males: 1.54 (0.56) pg/ml; hs-CRP females: 1.67 (3.15) mg/L; males: 1.27 (2.47) mg/L). Univariate analyses confirmed that IL-6 and hs-CRP levels as well as TICS and PSS scores did not differ between cohorts at T0.

### IL-6 change

The planned LMM revealed no significant module x time x sex or module x time interaction effect, and a trend-level module x sex interaction effect (*F*(3,537) = 2.35, *p* = 0.072, ω^2^ = 0.012). Thus, no follow-up contrasts were conducted. There was, however, a significant effect of baseline IL-6 concentration (*F*(1,383) = 20.8, *p* < 0.001, ω^2^ = 0.041). One reason for this pattern of results may be that some, but not all participants responded to the training, leading to a trend-level effect. We therefore explored in a second LMM whether participants with higher inflammatory load at baseline benefitted more from the training. To this end, the IL-6-baseline variable was interacted with the module x sex interaction term and its components, because the trend-level module x sex interaction suggested latent sex differences, which may become more pronounced as a function of baseline inflammation. The results showed only a trend-level interaction of sex x module x IL-6-baseline (*F*(3, 464) = 2.38, *p* = 0.069, ω^2^ = 0.010), but a significant interaction of module x IL-6-baseline (*F*(3, 462) = 5.33, *p* = 0.001, ω^2^ = 0.063).

**Figure 2:**
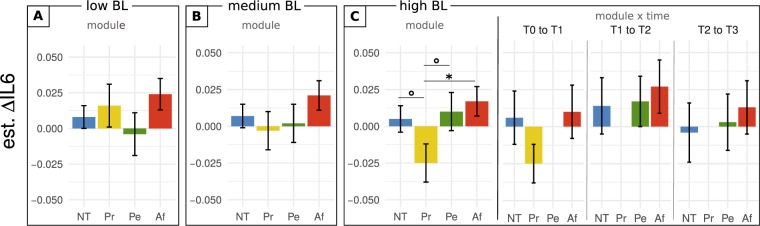
IL-6 change as a function of training module and baseline inflammation. The effect of baseline IL-6 concentration is illustrated through model predictions of IL-6 change when setting baseline concentration to low, medium, or high. The three baseline inflammation categories correspond to cut-off values from the 25^th^ (i.e., low baseline), 50^th^ (i.e., medium baseline), and 75^th^ (i.e., high baseline) percentile of the sample population. (**A,** **B**) estimated average change per module for low and medium baseline IL-6 levels, respectively. (**C**) estimated average change per module, and change per module and time point, for high baseline IL-6. Error bars represent +/− 1 SEM; ΔIL-6, change in ln-transformed interleukin-6; BL, baseline [inflammation]; NT, no training; Pr, Presence; Pe, Perspective; Af, Affect; °, trend at 0.05 < *p* < = 0.1; *significant at *p* <  = 0.05.

To visualize the pattern of the significant interaction, we extracted model estimates for the effects of training on participants with low, medium, and high baseline inflammation, by setting the IL-6-baseline variable to cut-off values from the 25^th^, 50^th^, and 75^th^ percentile of the sample population, respectively (Fig. 2). Estimates were also contrasted through follow-up pairwise *t*-tests. Since baseline inflammation was a continuous variable, these categorical contrasts primarily serve illustrative purposes. There were no significant differences in model estimates for participants with low or medium baseline inflammation. For those with high baseline inflammation, *t*-tests suggested that IL-6 values decreased significantly more after the Presence training than after Affect training (*t*(569) = 2.41, *p* = 0.016, est.: −0.042, 95% CI: −0.075, −0.008), and marginally more than after no training (*t*(645) = 1.88, *p* = 0.061) or Perspective training (*t*(597) = 1.80, *p* = 0.073).

To address the potential confound that training modules were practiced at different time points and with differing durations of prior training, estimates from the non-significant module x time interaction were also plotted for low, medium and high baseline inflammation and contrasted through *t*-tests. Although the results for high baseline inflammation showed greatest IL-6 decrease following Presence training, this change did not differ significantly from no training or Affect practiced between T0 and T1 (Fig. 2C). Change for medium and low baseline inflammation showed no significant differences, aligning with the average null findings (Supplementary Figure S1).

### hs-CRP change

The planned LMM revealed no significant module x time x sex or module x time interaction effect, but a trend-level module x sex interaction (*F*(3,578) = 2.30, *p* = 0.077, ω^2^ = 0.016), and a significant effect of baseline hs-CRP concentration (*F*(1,342) = 40.0, *p* < 0.001, ω^2^ = 0.066), mirroring IL-6 results. Again, no follow-up contrasts were conducted. In line with the analyses of IL-6, we explored the interaction of the hs-CRP-baseline variable with the trend level module x sex interaction, as well as with module and sex alone. The analysis revealed a significant interaction of module x sex x hs-CRP-baseline (*F*(3,476) = 4.59, *p* = 0.004, ω^2^ = 0.010). Since the original term of interest, module, was part of a significant interaction, lower order effects of module that would average over both sexes were not considered further.

**Figure 3:**
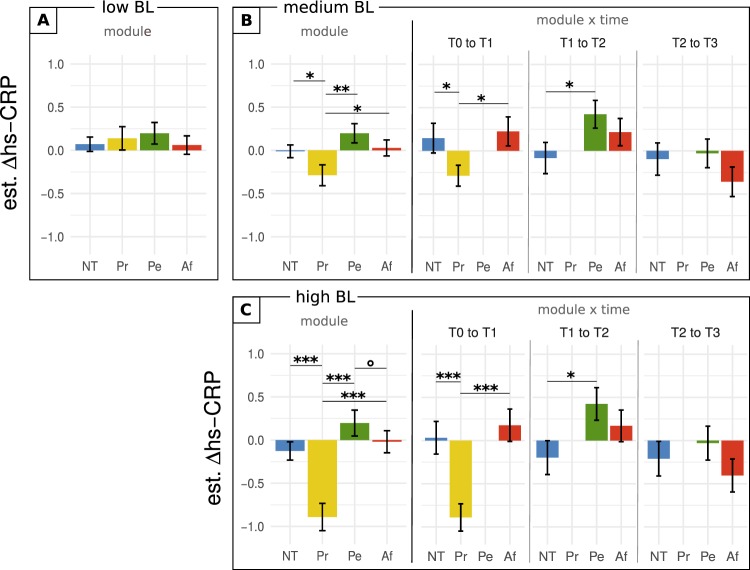
hs-CRP change in males as a function of training module and baseline inflammation. The effect of baseline hs-CRP concentration in males is illustrated through model predictions of hs-CRP change for low, medium, or high baseline concentration (25^th^, 50^th^, and 75^th^ percentile of the sample population). (**A**) estimated average change per module for low baseline hs-CRP levels. (**B,C**) estimated average change per module, and change per module and time point, for medium and high baseline hs-CPR, repectively. Error bars represent + /− 1 SEM; Δhs-CRP, change in ln-transformed high sensitive C-reactive protein; BL, baseline [inflammation]; NT, no training; Pr, Presence; Pe, Perspective; Af, Affect; °, trend at 0.05 < p < = 0.1; *significant at *p* <  = 0.05; **significant at *p* < = 0.01; ***significant at *p* <  = 0.001.

**Figure 4:**
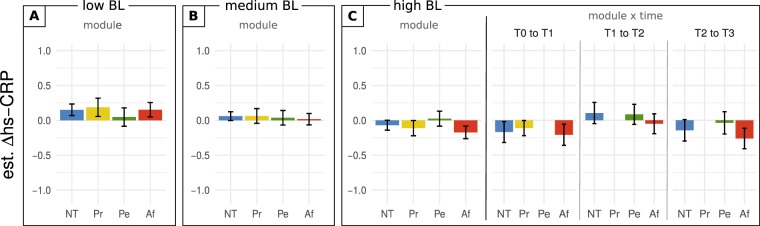
hs-CRP change in females as a function of training module and baseline inflammation. The effect of baseline hs-CRP concentration in females is illustrated through model predictions of hs-CRP change for low, medium, or high baseline concentration (25^th^, 50^th^, and 75^th^ percentile of the sample population). (**A**,**B**) estimated average change per module for low and medium baseline hs-CRP levels, respectively. (**C**) estimated average change per module, and change per module and time point, for high baseline hs-CRP. Error bars represent +/− 1 SEM; Δhs-CRP, change in ln-transformed high sensitive C-reactive protein; BL, baseline [inflammation]; NT, no training; Pr, Presence; Pe, Perspective; Af, Affect.

To decompose the significant interaction, the model estimated training effects for males and females with low, medium, and high baseline hs-CRP inflammation were extracted (Figs. 3, 4), following the same procedure as for IL-6. As before, estimates were contrasted through *t*-tests for illustrative purposes. There were no significant differences in model estimates for males with low baseline inflammation. For males with medium and high baseline inflammation, estimated hs-CRP values decreased significantly more after Presence training than after all other modules (medium inflammation, no training: *t*(595) = 1.96, *p* = 0.05, est.: −0.278, 95%CI: −0.556, 0.0; Affect: *t*(563) = 1.98, *p* = 0.049, est.: −0.316, 95%CI: −0.631, −0.002; Perspective: *t*(601) = 2.77, *p* = 0.006, est.: −0.486, 95%CI: −0.830, −0.142; high inflammation, no training: *t*(643) = 4.05, *p* < 0.001, est.: −0.765, 95%CI: −1.137, −0.393, Affect: *t*(632) = 4.17, *p* < 0.001, est.: −0.872, 95%CI: −1.281, −0.462; Perspective: *t*(626) = 4.55, *p* < 0.001, est.: −1.087, 95%CI: −1.558, −0.617). In addition, there was a trend level difference between Perspective and no training effects on males with high baseline inflammation (*t*(578) = 1.76, *p* = 0.079).

Just as for IL-6, estimates from the module x time x baseline inflammation interaction were plotted to investigate the consistency of effects that were significant on average, and the impact of different total training durations (Fig. 3B,C, and Supplementary Figure S2). For males with high or medium baseline inflammation, *t*-test showed that Presence remained associated with a significantly greater reduction in hs-CRP than no training or Affect practiced between T0 and T1 (medium inflammation, no training: *t*(498) = 2.07, *p* = 0.039, est.: −0.435, 95%CI: −0.023, −0.847; Affect: *t*(496) = 2.48, *p* = 0.014, est.: −0.513, 95%CI: −0.920, −0.106; high inflammation, no training: *t*(603) = 3.76, *p* < 0.001, est.: −0.922, 95%CI: −1.40, −0.441; Affect: *t*(580) = 4.34, *p* < 0.01, est.: −1.07, 95%CI: −1.55, −0.585). Additionally, Perspective and no training effects differed significantly at T1 to T2 (medium inflammation: *t*(592) = 2.10, *p* = 0.036; est.: 0.508, 95%CI: 0.983, 0.033; high inflammation: *t*(633) = 2.30, *p* = 0.022; est.: 0.622, 95%CI: 0.09, 1.15), but not at T2 to T3.

For females, none of the contrasts revealed a significant difference (Fig. 4). For the sake of completeness, the module by time estimates are nonetheless provided (high baseline inflammation shown in Fig. 4C, low and medium in Figure S2).

In sum, specifically the effect of the Presence training varied as a function of baseline inflammation, and was associated with stronger decreases the higher individuals’ baseline inflammation. In IL-6 this effect was visible on average, and in hs-CRP also when comparing module effects with matched durations of prior training, but only in males.

### Psychological and behavioral variables of interest

Finally, we investigated if the observed effects of training may have been the result of change in experienced stress (measured via TICS and PSS scores), and if they were affected by practice frequency or change in health-related behavior. The influence of all of these variables was evaluated through two separate regression analyses, one for each of the exploratory results, i.e., the Presence effect on IL-6, and on hs-CRP in males. The regression analyses revealed no significant effect of subjectively reported stress, behavioral change, or practice frequency on either outcome (Bonferroni corrected *p*-values for multiple testing across the two outcomes). Supplementary Table S5 describes the corrected and uncorrected results in detail.

## Discussion

The present study investigated how three distinct contemplative mental training techniques influenced basal Interleukin-6 (IL-6) and high-sensitive C-reactive protein (hs-CRP) levels in 298 healthy, low-risk adults. The modules Presence, Affect, and Perspective cultivated attention-based, socio-affective, and socio-cognitive capacities, respectively. None of the three investigated types of mental practice significantly affected circulating levels of IL-6 or hs-CRP on a group level. However, exploratory follow-up analyses indicated that Presence training significantly reduced biomarker levels in participants with relatively higher inflammatory levels at baseline. This pattern of results suggests a floor effect in our healthy sample, in that the majority of participants had such low basal levels of IL-6 and hs-CRP that they could not be further reduced. Our overall null finding is therefore not at odds with previous evidence of reduction in patients and at-risk populations, but aligns with the notion that low-risk individuals may be less likely to experience positive biological health outcomes from contemplative mental training^6^. Different and larger effects of mental training may be observed even in healthy individuals when examining a challenged immune system.

Exploratory analyses identified a selective effect of Presence training in reducing IL-6 and hs-CRP levels, which was the stronger the higher participants’ inflammatory load was at baseline. In IL-6 this outcome was only significant when contrasting average module effects, although Presence continued to be associated with the largest numerical IL-6 reductions compared to no training and Affect training practiced at the same time interval. In hs-CRP, Presence-related reduction was only observable in males, and remained significant when contrasting module effects with matched training durations. Providing one explanation for the stronger training effect on hs-CRP levels, the acute phase protein hs-CRP is released downstream from IL-6 and has a more specific pro-inflammatory role, which could make it a more reliable indicator of low-grade inflammation.

The Presence module trained classic present-moment attention practices similar to established mindfulness-based training protocols that have been associated with reductions in basal IL-6 or hs-CRP in patient and at-risk populations^16–20^. Our finding therefore generally aligns with these outcomes. In addition, the coinciding decrease in IL-6 and hs-CRP following Presence makes it less likely that this exploratory result reflects a false positive. Conversely, the Affect module, which had similarities with compassion-based training protocols, was not associated with reductions in IL-6 or CRP. To date, investigations on the effects of compassion-based training are sparse, but our result contradicts one finding of associations between basal CRP reduction and amount of engagement in compassion-based training^36^. Overall, our data suggests that in MBSR programs, which combine different practice types, attention-based training may be the most effective component for achieving a reduction in inflammatory markers. Replication of this exploratory outcome is necessary, however.

We suggest that the increases in IL-6 and hsCRP concentrations we observed after some instances of the Perspective or Affect modules, although seldom significant and inconsistent across the two biomarkers and multiple practice instances, may have occurred as a result of the strains associated with engaging in some types of mental practice. We previously found that the core meditation practices of Affect and Perspective were associated with higher subjective and physiological measures of effort and arousal than the Presence Breathing Meditation^51^, which may explain the observed pattern.

In contrast to our hypotheses, the identified IL-6 and hs-CRP reductions were not related to change in chronic stress as measured through self-reports. It should be noted that a dissociation between questionnaire-based and biological data as found here is not uncommon; in previous work we detected such methodological clustering already at study baseline^52^. Associations between inflammatory markers and subjectively perceived stress may be more readily observable in extreme groups, such as chronically stressed caregivers. For the present sample, there are alternative pathways that could also underlie the observed effect. For example, Presence trained the regulation of attention-based processes. Since it has been proposed that the immune system is implicated in self-regulatory mechanisms^53^, training self-regulation may, in turn, improve regulation of the immune system.

In additional secondary analyses, IL-6 and hs-CRP change was not linked to change in the health-related behavioral variables physical exercise, sleep, and healthy diet, suggesting that the observed change in inflammation was due to the mental training rather than behavioral adjustments. Training compliance did not relate to the strength of observed effects. We attribute this lack of a practice effect to the overall high compliance of our participants, leading to little variation in practice times.

Training-related reductions in hs-CRP, but not IL-6, were specific to male participants. Since both biomarkers were employed to measure the same construct - chronic low-grade inflammation - this pattern likely indicates that training effects were more robust for male than female participants. Several processes could be implicated in the observed sex-differences. Sex steroids have been found to influence IL-6^54^ and hs-CRP concentrations^55,56^, and can cause elevated and more variable distributions of IL-6 and hs-CRP, similar to the pattern we detected at study baseline. Hormone-related fluctuations may, for example, have distorted the classification of baseline inflammatory load in female participants, leading to less accurate estimates of how training affected females compared to males with relatively higher inflammatory load. Beyond biological influences, speculatively, differences in socialisation and associated psychosocial factors may also play a role in the effects of training. For example, men and women reportedly employ different coping strategies during emotion regulation^57^, and experience different types of prominent stressors^58^. It is possible that the Presence training cultivated coping strategies that were more appealing to male participants, or more effective towards their stressors, resulting in a more robust effect. Since we did not hypothesize a three-way interaction of training module, participant sex, and baseline inflammation a-priori, results of our exploratory analyses should be viewed with some caution and require replication in future studies. Since mental training outcomes are overall inconsistent^8^, it would indeed be interesting to investigate whether more homogeneous results can be found by differentiating between the sexes.

There are several limitations to the present study. Due to the considerable effort associated with organizing and conducting each of the training modules, we could not fully counterbalance the module sequences. The comparability of modules at the final time point was therefore compromised by the divergence in preceding practice styles (either Presence and Affect, or Presence and Perspective). Furthermore, we did not track female hormonal cycle in naturally cycling women, or whether participants suffered from minor diseases or infections at the time of testing. Both variables can influence the assayed inflammatory biomarkers and add noise to the dependent variables. Finally, the self-report measures on health-related behavior were only subjective assessments, which are vulnerable to bias. Objectively tracking change in physical exercise, diet, and sleep may have yielded different results.

In sum, we find that in healthy adults, contemplative mental training predominantly reduces inflammatory biomarkers in individuals suffering from signs of a strained or overactive immune system. Mental training was ineffective in low-risk individuals. For the subgroup of participants showing change in biomarkers over time, only the training of present-moment focused attention was effective. This training effect was more consistent for men than women. Our findings thus suggest that health effects of contemplative mental training should be investigated in consideration of individual differences, including participant sex and initial health condition, such as pro-inflammatory status.

## Supplementary information

Supplementary material

## Data Availability

In line with new data regulations (General Data Protection Regulation, GDPR), we regret that our data cannot be shared publicly because we did not obtain explicit participant agreement for data-sharing with parties outside the Max Planck Institute for Human Cognitive and Brain Sciences (MPI CBS). The present work is based on personal data (age, sex and medical data) that could be matched to individuals. The data is therefore pseudonominized rather than anonymized and falls under the GDPR. Data are available upon request (contact via puhlmann@cbs.mpg.de).
