# Supplementary material for: Only vulnerable adults show change in chronic low-grade inflammation after contemplative mental training: evidence from a randomized clinical trial
Source: Sci Rep. 2019 Dec 18;9:19323. doi: 10.1038/s41598-019-55250-3 (PMC6920474; doi:10.1038/s41598-019-55250-3)
Supplement: Supplementary file 1 — Supplementary material [file 41598_2019_55250_MOESM1_ESM.pdf]

Supplementary information

for

Only vulnerable adults show change in chronic low-grade inflammation after contemplative mental training: evidence from a randomized clinical trial

Puhlmann, L.M.C.[*], Engert, V., Apostolakou, F., Papassotiriou, I., Chrousos, G.P., Vrtička, P.[a], Singer, T.[a]

[*]Corresponding author

[a]These authors jointly supervised this work

***Figure S1.* IL-6 change per timepoint and module.**

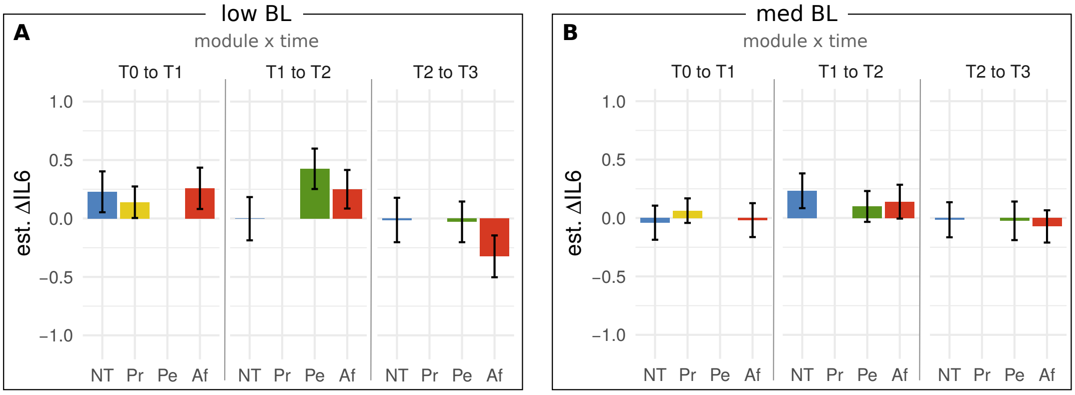

Notes: Bar plots show estimated average IL-6 change as a function of timepoint and training module, given low (panel **A**) or medium (panel **B**) baseline concentration of IL-6 (corresponding to 25th and 50th percentile of the sample population, respectively). Error bars represent +/- 1 SEM; ΔIL-6, change in ln-transformed interleukin-6; BL, baseline [inflammation]; NT, no training; Pr, Presence; Pe, Perspective; Af, Affect.

Figure S2. **hs-CRP change per timepoint, module and sex.**

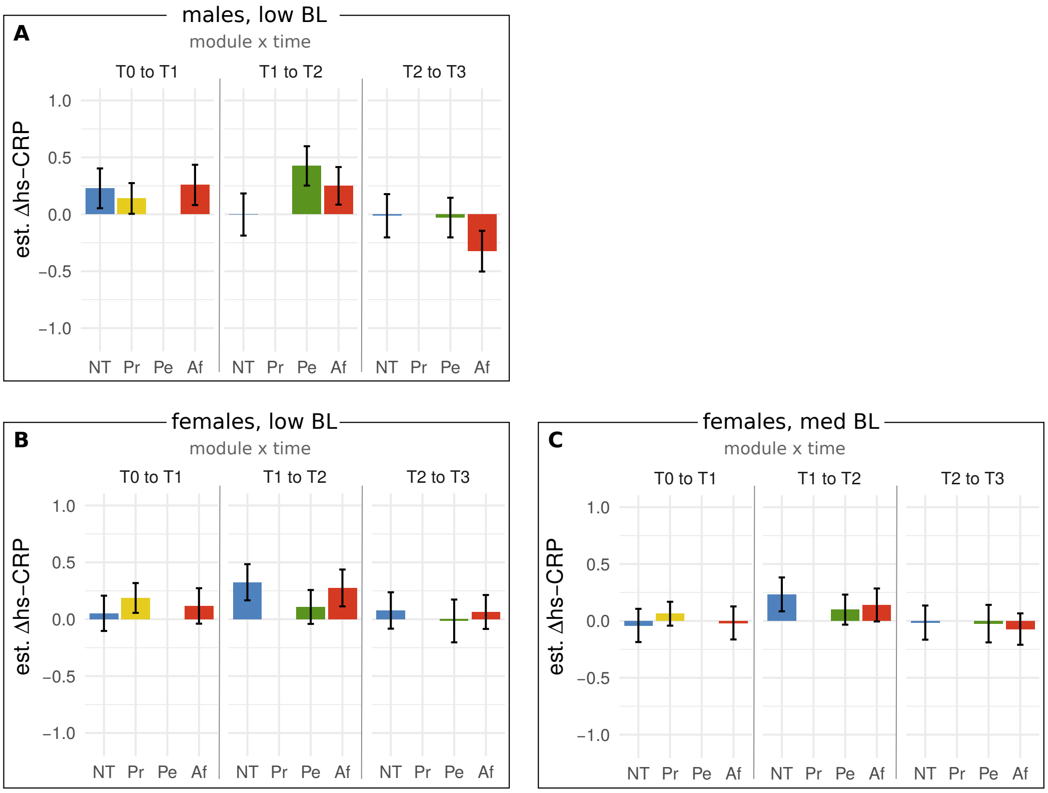

Notes: Bar plots show estimated hs-CRP change as a function of timepoint, training module and participant sex, given low (panels **A, B**) or medium (panel **B**) baseline concentration of hs-CRP (corresponding to 25th and 50th percentile of the sample population, respectively). Error bars represent +/- 1 SEM; Δhs-CRP, change in high sensitive C-reactive protein; BL, baseline [inflammation]; NT, no training; Pr, Presence; Pe, Perspective; Af, Affect.

Table S1. **Availability of raw data and reasons for missing cases.**

Variable	Available N (raw data)	Reasons for missing cases
**Age**	332	N/A
**BMI**	317	Study dropout (N = 2)<br>Study exclusion (N = 2)<br>No BMI assessed (N = 11)
**Sex**	332	N/A
**IL-6/ hs-CRP**	**T0** N = 315	Study dropout (N = 2)<br>Study exclusion (N = 2)<br>No blood sampling (exclusion) (N = 3)<br>No blood sampling (missing) (N = 10)
	**T1** N = 298	Study dropout (N = 12)<br>No blood sampling (missing) (N = 15)
	**T2** N = 213	Study dropout (N = 5)<br>TC3 Study completed (N = 77)[1)]<br>No blood sampling (missing) (N = 18)
	**T3** N = 216	Study dropout (N = 7)<br>No blood sampling (missing) (N = 8)
**TICS, PSS**	**T0**<br>N = 322	Study dropout (N = 2)<br>Study exclusion (N = 2)<br>No questionnaire data (N = 6)
	**T1**<br>N = 311	Study dropout (N = 12)<br>No questionnaire data (N = 5)
	**T2**<br>N = 232 (TICS)<br>N = 233 (PSS)	Study dropout (N = 5)<br>TC3 Study completed (N = 77)[1)]<br>No questionnaire data (TICS: N =2, PSS: N = 1)
	**T3**<br>N = 225 (TICS)<br>N = 226 (PSS)	Study dropout (N = 7)<br>No questionnaire data (TICS: N = 2; PSS: N = 1)

Note: TP denotes time point; BMI, body mass index; IL-6, interleukin-6; hs-CRP, high sensitive C-reactive protein; TICS, Trier Inventory for Chronic Stress; PSS, Perceived Stress Scale.

[1)] N = 4 prev. dropout from TC3

Table S2. **Availability of change scores and reasons for missing cases.**

Variable	Available N<br>(per TP)	Reasons for missing cases
**IL-6 / hs-CRP (change)**	**T0 to T1**<br>N = 291	Study dropout, T0 & T1 (N = 14)<br>Study exclusion (N = 2)<br>No blood sampling (exclusion) (N = 3)<br>No blood sampling at T0 or T1 (N = 22)
	**T1 to T2**<br>N = 210	Study dropout T2 (N = 5)<br>TC3 Study completed (N = 77)[2)]<br>No blood sampling at T1 or T2 (N = 21)
	**T2 to T3**<br>N = 203	Study dropout T3 (N = 7)<br>No blood sampling at T2 or T3 (N = 21)
**TICS, PSS (change)**	**T0 to T1**<br>N = 309	Study dropout, T0 & T1 (N = 14)<br>Study exclusion (N = 2)<br>No questionnaire data T0 or T1 (N = 7)
	**T1 to T2**<br>N = 229 (TICS)<br>N = 230 (PSS)	Study dropout T2 (N = 5)<br>TC3 Study completed (N = 77)[2)]<br>No questionnaire data T1 or T2 (TICS: N = 5, PSS: N = 4)
	**T2 to T3**<br>N = 224 (TICS)<br>N = 225 (PSS)	Study dropout T3 (N = 7)<br>No questionnaire data T2 or T3 (TICS: N = 3, PSS: N = 2)
**Physical exercise, Healthy diet, Sleep**[1)]	**T0 to T1**<br>N = 220<br>(sleep N= 219)	**Only assessed in TCs (N = 242)**<br>TCs Study dropout T1 (N = 10)<br>TCs Study exclusion (N = 0)<br>No questionnaire data T1 (N = 12, sleep N = 13)
	**T1 to T2**<br>N = 145<br>(sleep N= 144)	TCs Study dropout T2 (N = 3)<br>TC3 Study completed (N = 77)[2)]<br>No questionnaire data T2 (N = 7, sleep N = 8)
	**T2 to T3**<br>N = 140<br>(diet N = 141)	TCs Study dropout T3 (N = 4)<br>No questionnaire data T3 (N = 8, diet = 7)
**Practice frequency**[1)]	**T0 to T1**<br>N = 226 (med)<br>N = 74 (dyad)*	**Only assessed in TCs (N = 242)**<br>TCs Study dropout T1 (N = 10; 4 from TC3)<br>TCs Study exclusion (N = 0)<br>No practice data T1 (N = 6, 3 from TC3)<br>*only TC3 completed a module with dyads at T0-T1
	**T1 to T2**<br>N = 149 (both)	TCs Study dropout T2 (N = 3)<br>TC3 Study completed (N = 77)[2)]<br>No practice data T2 (N = 3)
	**T2 to T3**<br>N = 145 (both)	TCs Study dropout T3 (N = 4)<br>No practice data T3 (N = 3)

Note: TP denotes time point; TC, training cohort; IL-6, interleukin-6; hs-CRP, high sensitive C-reactive protein; TICS, Trier Inventory for Chronic Stress; PSS, Perceived Stress Scale.

[1)] not including data from drop outs of the present study (i.e. participants without IL.6, hs-CRP, TICS and PSS data)

[2)] N = 4 prev. dropout from TC3. Although N = 77 participants of the TC3 successfully completed the trial, due to N = 5 missings at T0 or T1 there are N = 72 complete change scores for the TC3.

Table S3. **Mean (SD) raw change in biomarkers and self-report questionnaires.**

		T0 to T1	T1 to T2	T2 to T3
**No Training**	**IL-6**	-0.071 (0.07)	0.014 (0.02)	0.024 (0.04)
	**hs-CRP**	0.079 (0.22)	0.089 (0.30)	0.007 (0.25)
	**TICS**	-1.66 (4.81)	0.54 (5.16)	-0.17 (4.28)
	**PSS**	-0.80 (5.06)	0.12 (4.62)	-0.08 (3.77)
**Presence**	**IL-6**	-0.037 (.03)		
	**hs-CRP**	-0.063 (.18)		
	**TICS**	-1.46 (5.39)		
	**PSS**	-0.33 (5.39)		
**Affect**	**IL-6**	0.068 (.08)	0.069 (0.04)	0.023 (0.07)
	**hs-CRP**	0.094 (.37)	0.160 (0.21)	-0.613 (0.31)
	**TICS**	-0.55 (5.23)	0.03 (5.03)	0.01 (4.62)
	**PSS**	-0.99 (5.07)	0.11 (5.53)	0.37 (5.16)
**Perspective**	**IL-6**		0.026 (0.09)	0.017 (0.07)
	**hs-CRP**		0.284 (0.39)	-0.104 (0.23)
	**TICS**		-0.16 (5.50)	-2.19 (5.95)
	**PSS**		-1.62 (5.83)	-2.55 (5.93)

Note: Change scores per time point and module were calculated by taking the difference between raw scores from each set of consecutive time points (T1 minus T0, T2 minus T1, and T3 minus T2). IL-6 denotes interleukin-6; hs-CRP, high sensitive C-reactive protein; TICS, Trier Inventory for Chronic Stress; PSS, Perceived Stress Scale; SD, standard deviation.

***Table S4.* Pearson correlation matrix of all measures available at baseline (T0)**.

	**Age**	**BMI**	**IL-6** (ln)	**hs-CRP** (ln)	**TICS**	**PSS**
**Age**	-	.273**	.007	.244**	-.076	-.062
**BMI**		-	.102	.310**	.014	-.028
**IL-6** (ln)			-	.252**	.049	.013
**hs-CRP** (ln)				-	-.044	-.065
**TICS**					-	.685**
**PSS**						-

Note: Additional partial correlations controlling for age, BMI and sex showed that IL-6 and hs-CRP levels were correlated at T0 (r=.222, p <.001), but neither marker correlated with baseline TICS or PSS scores. BMI denotes body mass index; IL-6, interleukin-6; hs-CRP, high sensitive C-reactive protein; TICS, Trier Inventory for Chronic Stress; PSS, Perceived Stress Scale. **: significant with p<.01.; *: significant with p<.05.

Table S5. **Follow-up regression analyses.**

**A**	**Beta**	**t-value**	**p-value** (uncor.)	**p-value** (cor.)
**Age**	.148	1.95	.053	.106
**BMI**	.361	4.75	<.001**	<.001**
**Sex**	-.228	-3.10	.002**	.004**
**IL-6 baseline**	-.591	-7.75	<.001**	<.001**
**TICS change**	.124	-1.56	.12	-
**PSS change**	-.093	-1.15	.25	-
**Meditation pract.**	-.088	-1.24	.22	-
**Physical exercise**	.043	0.58	.56	-
**Healthy diet**	-.015	-0.19	.85	-
**Sleep**	.081	1.17	.25	-

**B**	**Beta**	**t-value**	**p-value** (uncor.)	**p-value** (cor.)
**Age**	.126	0.94	.35	-
**BMI**	.192	1.47	.15	-
**hsCRP baseline**	-.605	-4.44	<.001**	<.001**
**TICS change**	-.059	-0.45	.66	-
**PSS change**	.056	0.42	.68	-
**Meditation pract.**	-.023	-0.18	.86	-
**Physical exercise**	.097	0.78	.44	-
**Healthy diet**	-.183	-1.46	.15	-
**Sleep**	.003	0.03	.98	-

Note: Regression analyses were conducted to test for the role of several variables of interest within the observed training effects. **A**, effect of Presence on IL-6 (overall model result: $F(10, 125)=8.39$, $p<.001$, corr. $p<.001$); **B**, effect of Presence on hs-CRP in males (overall model result: $F(9, 45)=2.73$, $p=.013$, corr. $p=.026$). Beta coefficients are standardized. *P*-values are reported uncorrected ("uncor.") and, if significant, Bonferroni corrected ("cor."). IL-6 denotes interleukin-6; hs-CRP, high sensitive C-reactive protein; TICS, Trier Inventory for Chronic Stress; PSS, Perceived Stress Scale. **: significant with $p<.01$.; *: significant with $p<.05$.
